# Two-Dimensional Graphene-Based Potassium Channels Built at an Oil/Water Interface

**DOI:** 10.3390/ma16155393

**Published:** 2023-07-31

**Authors:** Xiaoyuan Wang, Hanhan Yang, Zhenmei Yu, Zengtao Zhang, Yong Chen

**Affiliations:** School of Chemical and Environmental Engineering, Shanghai Institute of Technology, Shanghai 201418, China

**Keywords:** graphene laminar membrane, oil/water interface, angstrom-scale, ion transfer, cation-π interaction

## Abstract

Graphene-based laminar membranes exhibit remarkable ion sieving properties, but their monovalent ion selectivity is still low and much less than the natural ion channels. Inspired by the elementary structure/function relationships of biological ion channels embedded in biomembranes, a new strategy is proposed herein to mimic biological K^+^ channels by using the graphene laminar membrane (GLM) composed of two-dimensional (2D) angstrom(Å)-scale channels to support a simple model of semi-biomembrane, namely oil/water (O/W) interface. It is found that K^+^ is strongly preferred over Na^+^ and Li^+^ for transferring across the GLM-supported water/1,2-dichloroethane (W/DCE) interface within the same potential window (-0.1-0.6 V), although the monovalent ion selectivity of GLM under the aqueous solution is still low (K^+^/Na^+^~1.11 and K^+^/Li^+^~1.35). Moreover, the voltammetric responses corresponding to the ion transfer of NH_4_^+^ observed at the GLM-supported W/DCE interface also show that NH_4_^+^ can often pass through the biological K^+^ channels due to their comparable hydration–free energies and cation-π interactions. The underlying mechanism of as-observed K^+^ selective voltammetric responses is discussed and found to be consistent with the energy balance of cationic partial-dehydration (energetic costs) and cation-π interaction (energetic gains) as involved in biological K^+^ channels.

## 1. Introduction

Ultrahigh ion selectivity for certain ions is one of the most prodigious ion transport properties of intelligent biological ion channels embedded in cellular membranes [1,2,3]. One example is the most studied biological K^+^ channels. As reviewed by Kuang, biological K^+^ channels, existing in nearly all kingdoms of life, can achieve incredibly high ion selectivity for K^+^ over Li^+^ and Na^+^ (selectivity~10^4^), as well as remarkable ion permeation with near diffusion-limited rates (10^7^ ions channel^−1^s^−1^) [3]. Unfortunately, biological ion channels suffer from low efficiency and instability in industrial environments completely different from those existing in living organisms, which severely limits their large-scale application [4,5,6]. Following the patterns of elementary structure/function relationships of biological ion channels, artificial membranes or devices composed of zero-dimensional (0D) to three-dimensional (3D) solid-state pores or channels with angstrom(Å) or nanometer(nm) scale dimensions approaching the size of those cations typically present in biofluids and seawater, such as K^+^, Na^+^ and Li^+^, have recently gained attention worldwide due to their potential applications in water purification, desalination, biosensing, and energy conversion, etc. [4,5,6,7,8,9,10]. 

Notably, the growth of 2D materials has brought new opportunities to construct 2D channels, also called 2D cavities, slits, or capillaries [7]. As reviewed recently by Jin et al. [5], Zhang et al. [6], and Geim [7], 2D Å-scale channels have become the attractive platforms for investigating mass transport under Å-scale confinement. Recently, graphene-based laminar membranes or devices composed of 2D Å-scale or nm-scale ion channels [8,9,10,11,12,13,14,15,16,17,18,19,20,21] have also become attractive platforms to mimic natural ion channels because they can provide not only ion dehydration effects [8,13,15,16,17] but also cation-π interaction [18,19,20,21], as involved in biological ion channels [1,2,22,23]. 

Unfortunately, it still seems to be difficult for graphene-based laminar membranes formed by layer-by-layer stacking of graphene or graphene oxide (GO) sheets to achieve high monovalent ion selectivity because such ions, especially K^+^, Na^+^, and Li^+^, exhibit similarities in some respects, including hydration energies and ion radii. So far, the K^+^/Na^+^ and K^+^/Li^+^ selectivities of those graphene-based laminar membranes have remained lower than 2.0 [5,6], which is much less than the ultrahigh monovalent ion selectivity (~10^4^) of natural K^+^ channels [2]. Noticeably, all previous studies on the ion permeation of graphene-based laminar membranes have been conducted under the aqueous solution [8,9,10,11,12,13,14,15,16,17,18,19], which is different from the environment of ion channels embedded in biomembranes because it contains a hydrophobic phase [1,2]. As for biological K^+^ channels, the effects of the lipid environment are also worth investigating because lipids not only provide a suitable environment for channels to fold but can also participate in their activation [3]. Therefore, it is necessary to further investigate the transmembrane behaviors of ions through graphene-based laminar membranes under a biomimetic environment, such as the oil/water (O/W) interface [24,25,26].

The O/W interface, also called liquid/liquid (L/L) interface, has been considered not only a simple model to mimic the soft interface between the hydrophilic exteriors and hydrophobic interiors of biomembranes [24,25,26] but also a useful platform to investigate the interface behaviors of 2D nanosheets [27,28] and the transmembrane behaviors of ions through porous membranes [29,30,31,32,33]. Until now, there have been few reports on ion transfer (IT) behaviors at the O/W interface supported by graphene-based laminar membranes. The conventional porous membranes used to support the O/W interface contain 1D channels and can obtain arrays of 1D nano-O/W interfaces [20]. Obviously, the graphene-based membranes containing graphene layers and 2D channels are distinctive from conventional porous membranes, which can be used to construct arrays of the 2D nano-O/W interface. Based on our previous work on the IT behaviors at the membrane-supported O/W interface [30,31,32,33], a new strategy is proposed to mimic biological K^+^ channels embedded in biomembranes by using a graphene laminar membrane (GLM) composed of 2D Å-scale channels to support an O/W interface. Moreover, the ion-transfer voltammetry (ITV) is first employed to investigate the transmembrane behaviors of ions through GLM with comparison to the measurements of ionic conductivity (*G*) because the ITV employed at the polarizable O/W interface has been proven as a very useful technique for investigating the transmembrane behaviors of ions under a biomimetic environment [24].

**Figure 1:**
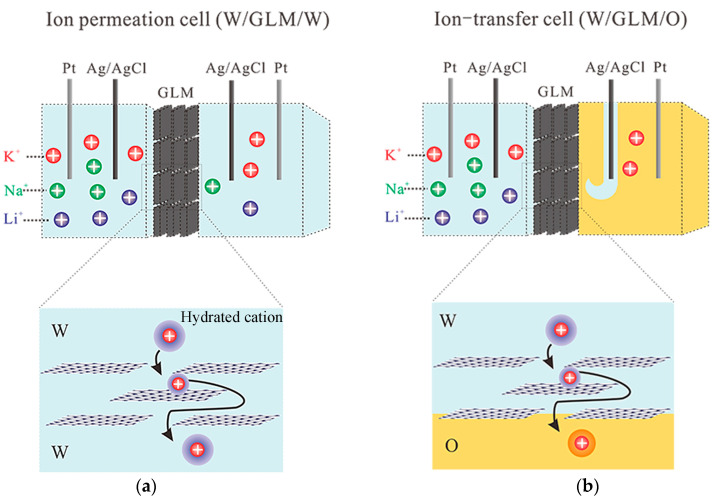
Schematic illustration for two kinds of transmembrane systems of small cations including Li^+^, Na^+^ and K^+^ through the graphene laminar membrane (GLM) composed of 2D Å-scale channels: (**a**) the W/GLM/W system in an ion permeation cell and (**b**) the W/GLM/O system in an ion-transfer cell.

Herein, a graphene laminar membrane (GLM) was fabricated by using a vapor filtration method with organic support, namely a porous polyethylene terephthalate (PET) membrane (Figure S1 in Supplementary Materials). PET membranes have been used as hard templates to synthesize nanoporous membranes and employed in the membrane-supported O/W interface due to their good resistance to organic solvents found in previous studies [30,31,32]. Detailed procedures employed in the fabrication of graphene laminar membranes by the vacuum filtration method are provided in the Supplementary Materials. Two kinds of systems for the investigation of transmembrane behaviors of Li^+^, Na^+^, and K^+^ are illustrated in Figure 1; namely, one of the aqueous phases (W) of the W/GLM/W system in a conventional ion permeation cell (Figure 1a) is replaced by an organic phase (O) to form the W/GLM/O system in an ion transfer cell (Figure 1b). The experimental conditions of systems of W/GLM/W and W/GLM/O are respectively described in the Supplementary Materials.

## 2. Results and Discussion

### 2.1. Microstructures of GLM/PET 

**Figure 2:**
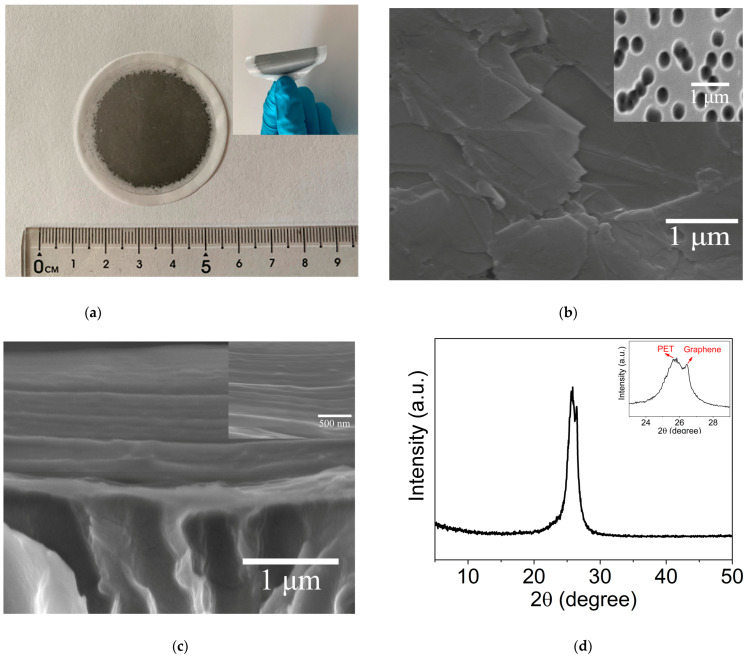
(**a**) Photographs of as-prepared GLM with PET support; (**b**) the top-view and (**c**) the side-view of SEM images of GLM; (**d**) the PXRD pattern of dry GLM. The inset in (**b**) is the SEM image of bare PET, and the inset in (**c**) is the corresponding SEM image of GLM with higher resolution, respectively.

The photograph of GLM formed on PET support was determined by optical microscopy. Figure 2a shows a photograph of an as-obtained GLM with a PET support, which demonstrates high flexibility without obvious damage. To further investigate the GLM, SEM was performed to examine the morphologies of GLM. Figure 2b,c show the top and side views of SEM images for the GLM formed on PET. It can be found that the as-prepared GLM consists of disordered arrays of flakes forming stacked laminar graphene sheets (Figure 2b) on the porous PET (the inset of Figure 2b). In addition, the side view of the SEM image (Figure 2c) clearly shows the laminar structure of GLM with a thickness of ~1.5 μm, illustrating the closely packed and mostly horizontal orientation of the restacked flakes, which is similar to those reports on the graphene-based laminar membranes prepared by using the vapor filtration method with other supports [14,18].

The PXRD pattern of dry GLM is shown in Figure 2d. Two characteristic diffraction peaks are presented at 25.5 and 26.5, which can be respectively assigned to the characteristic diffraction peak (25.5) of PET and the (002) peak (26.5) of the graphene laminar membrane [14]. Indeed, a characteristic diffraction peak is found at 25.5, assigned to the characteristic diffraction peak of PET (Figure S3a in Supplementary Materials). According to the Bragg equation [34], the *d*-spacing value of dry GLM is calculated to be about 0.34 nm, which accords with the previous report on the graphene laminar membrane with other support [14]. In addition, the (002) peak corresponding to the GLM in wet conditions (Figure S3b in Supplementary Materials) is almost identical to that of the dry GLM, which should be ascribed to the negligible swelling of graphene laminar membranes under aqueous solution [14].

### 2.2. Ion Transport through GLM in the System of W/GLM/W

**Figure 3:**
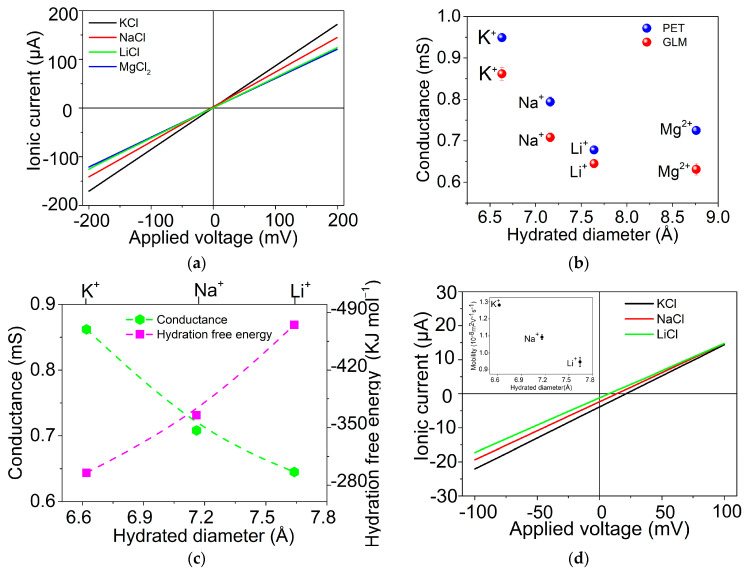
(**a**) *I–V* characteristics of GLM obtained in the W/GLM/W system, respectively, containing 0.1 M LiCl, 0.1 M NaCl, 0.1 M KCl, or 0.05 M MgCl_2_ aqueous solution; (**b**) ionic conductance (*G* = Δ*I*/Δ*V*) values of GLM and PET obtained from their corresponding *I-V* characteristics (Figure 3a and Figure S4), and the hydration diameters (*D_H_*) of cations are shown along the bottom *x* axis; (**c**) ionic conductance (*G*) as a function of *D_H_* and hydration free energy (ΔG_hyd_) of Li^+^, Na^+^, and K^+^; (**d**) *I-V* characteristics of GLM for KCl, NaCl, and LiCl aqueous solution under concentration ratio (10 mM/100 mM) and the ionic mobility values of Li^+^, Na^+^ and K^+^ as a function of their *D_H_* (the inset).

Typical current-voltage (*I-V*) characteristics of GLM obtained in the W/GLM/W system are shown in Figure 3a. The recorded characteristic *I-V* curves are found to be linear within the potential range of −0.2 V−0.2 V. The values of ionic conductance (*G*) of GLM measured by using different chloride salt are determined by fitting the slopes of the ionic current as a function of the applied voltage (*G* = Δ*I*/Δ*V*) [15]. As a comparison, the typical *I–V* characteristics for PET are shown in Figure S4 in Supplementary Materials, and the corresponding *G* values of PET and GLM are listed in Table 1. Obviously, the *G* values of bare PET are greater than those of GLM, indicating that the GLM formed on PET can impede the ion transport through GLM due to the effects of GLM on ion permeation.

According to the previous reports [14,15,16], the individual conductance of each cation is responsible for the differences among the total conductance of those chloride salt solutions, reflecting the permeability of different cations through the 2D Å-scale channels of GLM. As shown in Figure 3b, the relative transport rates of the cations are found to be K^+^ > Na^+^ > Li^+^ > Mg^2+^ on the basis of the calculated values of *G*, which is similar to the phenomena observed by using graphene laminar membrane or device composed of 2D Å-scale ion channels [14,15]. In addition, Figure 3c clearly demonstrates that the ionic conductivities and the hydration-free energies (ΔG_hyd_) of cations are closely related to their hydrated diameter (*D_H_*). According to the previous reports on the ion permeation through 2D graphene Å-scale channels [13,14,15,16], when their effective channel size approaches the hydration diameter of small ions, the hydration shells of hydrated ions have to acculturate to the Å-scale confinement by losing some water molecules. These molecules are accommodated inside the channels, leading to the partial dehydration of hydrated ions at the entry that impedes the ionic conductance significantly, namely the dehydration-related ion permeation mechanism under the Å-scale confinement [13,16]. If such a dehydration-related ion permeation mechanism is true, it can be inferred that the transport rates of two cations should be close as long as they have similar values of *D_H_* and ΔG_hyd_, such as K^+^ and NH_4_^+^. Indeed, the *G* values of GLM obtained from the characteristic *I–V* curves for the permeation of K^+^ and NH + 4are 0.862 ± 0.011 and 0.848 ± 0.007 mS (see Table 1 and Figure S5 in Supplementary Materials). These values indicate that the transport rates of K^+^ and NH_4_^+^ through GLM are almost the same due to the dehydration-related ion permeation mechanism under the Å-scale confinement, as discussed above.

In addition, the drift-diffusion experiment was also conducted by using two reservoirs filled with LiCl, NaCl, and KCl solutions in different concentrations [15]. As shown in Figure 3d, the *I–V* curves present a finite ion current, even in the absence of applied voltage and a negative current at 0 V. This implies that cations and anions should diffuse through GLM at different rates, namely the charge asymmetry effect in ion transport under the confinement of Å-scale channels [15,17]. According to the zero-current potential (*E_m_*) and the Henderson Equation [36], expressed as follows, we can calculate the mobilities (*μ^+^*) of three cations, the mobility (*μ^−^*) of Cl^−^, and their corresponding mobility ratios in GLM.

(1) $$\mu^{+}/\mu^{-}=-\frac{z_{+}}{z_{-}}\frac{\ln\left(∆\right)-z_{-}FE_{m}/RT}{\ln\left(∆\right)-z_{+}FE_{m}/RT}$$

(2) $$\sigma \approx F\left(c_{+}\mu^{+}+c_{-}\mu^{-}\right)$$

In Equation (1), *z_+_* and *z_−_* are the valence states of cations and anions, respectively; *F* is Faraday’s constant; *R* is the universal gas constant; *T = 300 K*; ∆ is the ratio of the concentration in the feed reservoir to that of the permeate reservoir, which is 10 in this experiment. In Equation (2), *σ* is the ionic conductivity value obtained from Figure 3d. The mobilities of Li^+^, Na^+^, K^+^, and Cl^−^ are respectively calculated as 0.95 ± 0.02 × 10^−8^, 1.15 ± 0.01 × 10^−8^, 1.28 ± 0.01 × 10^−8^, and 5.87 ± 0.14 × 10^−9^ m^2^ V^−1^ s^−1^. On one hand, the mobilities of cations are also related to their hydrated diameter (the inset of Figure 3d). On the other hand, the mobility ratios of K^+^/Cl^−^(2.18), Na^+^/Cl^−^(1.96), and Li^+^/Cl^−^(1.62) reflect the charge asymmetry effect in ion transport through Å-scale channels, as proposed by Geim et al. [15] and Wang et al. [17]; namely, an ion Cl^−^ can experience a remarkably larger friction force inside the Å-scale channel and, consequently, less mobility compared with cations. In contrast, the K^+^/Na^+^ and K^+^/Li^+^ selectivities of GLM are calculated as 1.11 and 1.35, respectively, according to their corresponding mobility ratios. These ratios are similar to the values reported previously [14,15,16] but still much lower than those of biological K^+^ channels [2], indicating that only Å-scale confinement is unlikely to provide high selectivity among Li^+^, Na^+,^ and K^+^ in the aqueous solution [15].

### 2.3. Ion Transfer across the GLM-Supported W/DCE Interface in the System of W/GLM/O

**Figure 4:**
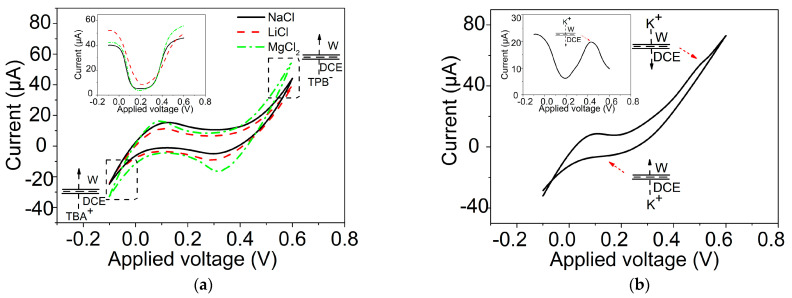

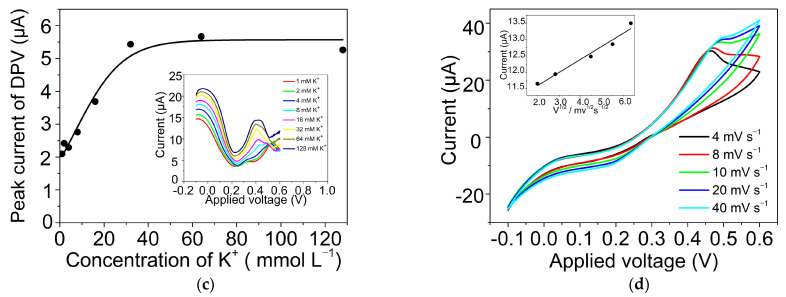
(**a**) CVs obtained at the GLM-supported W/DCE interface by using cell 1 (R is NaCl (*x* = 100), LiCl (*x* = 100), or MgCl_2_ (*x* = 50)) under a scan rate (*v*) of 50 mV s^−1^, and the insert is the corresponding DPVs; (**b**) CV obtained at the GLM-supported W/DCE interface by using cell 1 (*R* is KCl (*x* = 100)), and the insert is the corresponding DPV under a scan rate (*v*) of 50 mV s^−1^; (**c**) DPVs collected at the GLM-supported W/DCE interface for different concentrations of K^+^ by using cell 2 (*y* is 0 (blank experiment), 1, 2, 4, 8, 16, 32, 64, and 128 mM (from bottom to top)), and the inset is the corresponding plot of peak current (*I_p_*) for IT of K^+^ from W to DCE vs. (*C*(K^+^)) in aqueous solution; (**d**) CVs obtained at the GLM-supported W/DCE interface by using cell 1 (R is KCl (*x* = 100)) under different scan rates (4, 8, 10, 20, and 40 mV/s from inner to outside), and the inset is the relationship between *I_p_* and *v*^1/2^. Cells 1 and 2 employed herein are shown as Figure S2 in Supplementary Materials.

In order to preliminarily study ion transfer behaviors occurring at the O/W interface supported by a GLM in the W/GLM/O system (Figure 1b), water/1,2-dichloroethane (W/DCE) interface with extensive studies in the field of L/L interface electrochemistry [29,30,31,32,33] were adopted in this study. The ion transfer behaviors occurring at the GLM-supported W/DCE interface were investigated by using cyclic voltammetry (CV) and differential pulse voltammetry (DPV) [30,31,32,33]. Figure 4a shows the CV curves obtained at the GLM-supported W/DCE interface by using tetrabutylammonium tetraphenylborate (TBATPB) as the organic electrolyte and LiCl, NaCl, or MgCl_2_ as the aqueous electrolyte, presenting an extended potential window (−0.1–0.6 V) compared with that (0–0.42 V) obtained at the PET-supported W/DCE interface (Figure S6 in Supplementary Materials). As reported previously [33], the ion transfer of background electrolyte ions at the O/W interface, supported by the membrane with narrow channels, becomes more difficult due to the size effect of the narrow channels on the IT processes, which can lead to the extension of the potential window. Thus, the broader potential window obtained at the GLM-supported W/DCE interface demonstrates that the size effect of Å-scale channels of GLM can impede not only the ion transport behaviors in the W/GLM/W system, as mentioned above but also the ion transfer behaviors occurring at the O/W interface in the W/GLM/O system. However, no Faradaic response corresponding to the ion transfer can be observed within the extended potential window in the case of LiCl, NaCl, and MgCl_2_, which demonstrates that it is still difficult for Li^+^, Na^+^, and Mg^2+^ to transfer across the GLM-supported W/DCE interface. Moreover, the voltammetric results, obtained by using DPV with better resolution than CV [30,31,32,33], also show that there is not any Faradaic response appearing in the corresponding DPV curves of those cations (see the inset in Figure 4a).

Amazingly, when KCl is used as the aqueous electrolyte instead of LiCl, NaCl, or MgCl_2_, a well-defined and asymmetrically-shaped Faradaic response with the peak potential of 0.49 V appears within the same window (–0.1–0.6 V), as shown in Figure 4b. This result indicates that K^+^ could transfer across the GLM-supported W/DCE interface. Meanwhile, the DPV curve clearly presents the transfer wave of K^+^ from W to DCE at 0.45 V (see the inset of Figure 4b). To validate the as-observed DPV response ascribed to the IT of K^+^ from W to DCE, the changes of DPVs with the successive increase of K^+^ concentration (*C*(K^+^)) in the aqueous phase were further investigated. Figure 4c shows that the peak currents (*I_p_*) of DPV curves increase with *C*(K^+^), and the dependence of *I_p_* on *C*(K^+^) presents a good linear correlation within 1.0–31.2 mM (see the inset of Figure 4c), which illuminates that the transfer wave appearing in Figure 4b is, indeed, ascribed to the IT of K^+^ at the GLM-supported W/DCE interface. Additionally, Figure 4d demonstrates that the peak currents (*I_p_*) of CV curves for the IT of K^+^ from W to DCE increase with the scan rate (*v*) and display a linear dependence on v^1/2^ (the inset of Figure 4d). According to the Randles–Sevcik equation [30,31], the diffusion coefficient of K^+^ in the aqueous phase is calculated to be about 2.6 × 10^−9^ cm^2^ s^−1^. This is smaller than the value obtained at the W/DCE interface supported by the porous anodic aluminum (AAO) with nm-scale ion channels [29], indicating that the GLM with Å-scale ion channels could play a stronger block effect on the ion transport than the nanoporous AAO membrane.

**Figure 5:**
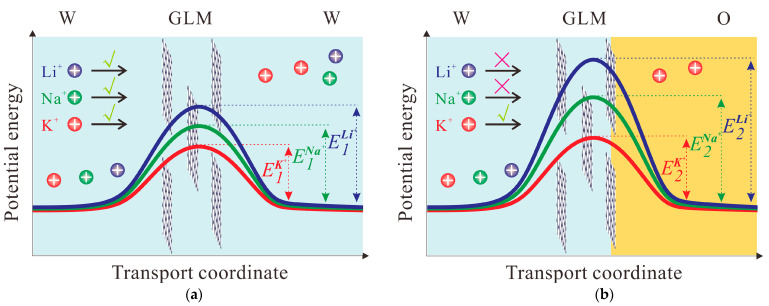
Schematic illustration for two kinds of systems with different energy barriers: (**a**) the W/GLM/W system in an ion permeation cell for the ion transport with energy barrier (*E*_1_), and (**b**) the W/GLM/O system in an ion-transfer cell for the ion transfer with energy barrier (*E*_2_). (K+ (red line), Na+ (green line), Na+ (blue line)).

All the above voltammetric results demonstrate that K^+^ is preferred over both Li^+^ and Na^+^ to transfer across GLM in the W/GLM/DCE system. In other words, such a GLM-supported W/DCE interface can reject Li^+^ and Na^+^ but exhibit selective ion transfer of K^+^ within the same potential window (–0.1–0.6 V). As shown in Figure 5a, it is expected that the energy barrier (*E*_1_) mainly induced by the partial dehydration (*E*_dh_) of hydrated ions should be the dominant effect on the ion transport through GLM as discussed above. However, the existence of an O/W interface behind GLM in the W/GLM/O system can act as a semi-biomembrane to produce an additional energy barrier besides *E*_dh_, that is, the Gibbs energy of ion transfer (ΔG_tr_) [24,25,26]. Therefore, the whole energy barrier (*E*_2_) for the transmembrane processes of ions in the W/GLM/O system should be higher than that of W/GLM/W system. As pointed out by Elimelech et al. [4], a small change in the energy barrier will significantly alter the solute selectivity due to the exponential dependence of solute permeability on the energy barrier (*E*). If the energy barrier (*E*_2_) for the transmembrane processes of K^+^ in the W/GLM/O system can be compensated more efficiently by the energetic gains derived from the ion-channel interactions, and if the external potential difference (Δ*V*) is applied as the driving force only enough for K^+^ to overcome its *E*_2_, namely, *E*_2_(K^+^) < Δ*V* < *E*_2_(Na^+^), *E*_2_(Li^+^), it will be possible to fulfill the selective ion transfer of K^+^ over Na^+^ and Li^+^ from W to O in the W/GLM/O system as shown as Figure 5b.

Normally, it is difficult for those strongly hydrophilic cations, including Li^+^, Na^+^, and K^+^, to directly transfer across the W/DCE interface within a limited potential window due to their high ΔG_tr_ [35,37,38,39]. According to our previous reports on the IT associated with the pre-dehydration process at the membrane-supported W/O interface [32,33], as-observed K^+^-selective voltammetric responses at the GLM-supported W/DCE interface should be closely related to the energy balance between the partial dehydration of hydrated ions (energetic cost) and the ion-channel interactions (energetic gain), such as the electrostatic interaction [33], as well as the well-known cation–π interactions between cations and the aromatic systems [22,40,41,42,43], including graphene-based nanosheets [20,42,43]. Moreover, the closely spaced binding sites of Å-scale channels can exert great repulsive force (F) between adjacent ions to facilitate the release of ions from the binding sites, resulting in a decreased energy barrier for ion transport, as demonstrated by Elimelech et al. [4].

**Figure 6:**
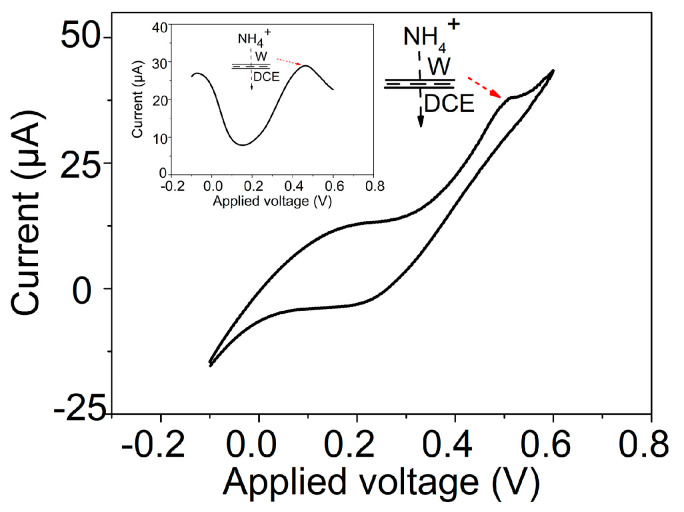
CV curve obtained at the GLM-supported W/DCE interface by using cell 1 (R is NH_4_Cl (*x* = 100)) under a scan rate (*v*) of 50 mV s^−1^ and the corresponding DPV curve (inset).

If such an energy balance mechanism as proposed herein is true, it could be possible for NH_4_^+^ to transfer across the GLM-supported W/DCE interface because NH_4_^+^ and K^+^ have similar values of ΔG_hyd_ and $E_{cation-\pi }$ (Table 1 and Table 2). As shown in Figure 6, the CV and DPV responses corresponding to the IT of NH_4_^+^ can also be observed at the GLM-supported W/DCE interface. Moreover, the peak potentials of CV and DPV curves for the IT of NH_4_^+^ (0.50 V and 0.46 V) are close to the values for the IT of K^+^ (0.49 V and 0.45 V). These values show that an energy balance between the partial-dehydration and cation-π interaction of NH_4_^+^ similar to that of K^+^ can also fulfill the transmembrane transfer of NH_4_^+^ across the GLM-supported W/DCE interface as it has been found that NH_4_^+^ can pass through the K^+^-channels in biological system [2,23]. Based on all the above discussion, as-observed K^+^-selective voltammetric responses at the GLM-supported W/DCE interface can be ascribed to the energy balance between the partial-dehydration and cation-π interactions of K^+^ within the Å-scale channels of GLM. These could play a key role in overcoming the energy barrier of the entire transmembrane process of K^+^ in the W/GLM/O system and accomplishing the ion transfer of K^+^ across the GLM-supported W/DCE interface. It has been found that a proper balance of the dehydration of ions (energetic cost) and the various interactions (energetic gain) between dehydrated K^+^ and channels, including the cation–π interactions between dehydrated K^+^ and several conserved aromatic residues in the pore region of K^+^-channel proteins, conducts K^+^ ions selectively across the cell membrane down the electrochemical gradient [1,2,22,23].

To the best of our knowledge, this study reveals for the first time that the 2D graphene channels under a biomimetic environment can act as K^+^ channels embedded inside the lipid bilayer of biomembranes to realize the desirable selective transmembrane transport of K^+^. Although precisely mimicking the complex and intelligent biological systems composed of ion-channel proteins and biomembranes, this work provides a new perspective. Based on the ion transfer across a membrane-supported O/W interface to understand more comprehensively the dehydration-related ion transport properties under the Å-scale confinement as involved in the natural ion channels, the results of this study will open a new path to further exploit the potential applications of those versatile laminar membranes in the fields of biomimetic ion channel and electrochemical ion sensing or extraction. In order to understand the mechanism of ion transfer and the structure-property-performance at the GLM-supported O/W interface more comprehensively, it is necessary to further investigate the transmembrane behaviors of some other cations at the membrane-supported O/W interface with comparison to the measurements of ionic conductivity by using different 2D laminar membranes, such as GO laminar membranes. In our opinion, GO or chemically modified GO laminar membranes may present another choice to achieve better ion permeation properties and a trade-off relationship between ion selectivity and ion transport flux in the aqueous solution and at the oil/water interface.

## 3. Conclusions

In this study, a novel strategy was applied to mimic K^+^ channels embedded inside biomembranes by using the graphene laminar membrane (GLM) composed of 2D graphene channels to support an O/W interface acting as a simple model of semi-biomembranes. In addition, the ion transfer voltammetry (ITV) was first employed to monitor the IT processes of some small monovalent cations, including Li^+^, Na^+^, K^+^, and NH_4_^+^ at the GLM-supported W/DCE interface with comparison to the measurements of ionic conductivity of those cations. Amazingly, K^+^ was greatly preferred over both Li^+^ and Na^+^ to transfer across the GLM-supported O/W interface. Moreover, the voltammetric responses for the IT of NH_4_^+^ at the GLM-supported W/DCE interface are also observed to often pass through the biological K^+^ channels due to the comparable dehydration energies and cation-p interactions of K^+^ and NH_4_^+^. The underlying mechanism of as-observed K^+^-selective voltammetric responses at the GLM-supported O/W interface is discussed and found to be consistent with the energy balance of cationic partial-dehydration (energetic cost) and hydrated cation-p interactions (energetic gain) as involved in K^+^ channel proteins. Our results first reveal that the 2D graphene-based Å-scale channels under a biomimetic O/W interface environment can play a similar role as K^+^ channel proteins embedded inside cellular membranes to realize the desirably selective transmembrane transport of K^+^. In our opinion, such a W/GLM/O system composed of 2D Å-scale channels and a biomimetic O/W interface will provide a useful platform to not only understand the ion discrimination of graphene channels from a new perspective by using versatile ion transfer voltammetries but also further exploit the potential applications of 2D laminar membranes in the fields of biomimetic ion channel and electrochemical ion sensing or extraction.

## Figures and Tables

**Table 1:** The hydrated diameter (*D_H_*) and hydration-free energy (ΔG_hyd_) of different cations and the ionic conductance (*G*) data obtained from their corresponding current-voltage (*I–V*) curves.

Ion	*D_H_* (nm)	ΔG_hyd_ (KJ/mol)	*G_GLM_* (mS)	*G_PET_* (mS)
K^+^	0.662 [35]	−295 [35]	0.862 ± 0.011	0.949 ± 0.003
Na^+^	0.716 [35]	−365 [35]	0.708 ± 0.008	0.794 ± 0.009
Li^+^	0.764 [35]	−475 [35]	0.645 ± 0.007	0.678 ± 0.004
Mg^2+^	0.876 [35]	−1830 [35]	0.631 ± 0.010	0.725 ± 0.002
NH_4_^+^	0.662 [35]	−285 [35]	0.848 ± 0.007	−

**Table 2:** The Gibbs energy of ion transfer (${\Delta G}^{O,W\to DCE}$) and the cation-π binding energy (*E*_cation-π_) of some monovalent ions (M^+^) with benzene or graphene (Gr).

Ion	${\Delta G}^{O,W\to DCE}\;\left(KJ/mol\right)$	*E*_cation-π_ (M^+^⋅⋅⋅C_6_H_6_) (KJ/mol)	*E*_cation-π_ (C_6_H_6_⋅⋅⋅M^+^⋅⋅⋅C_6_H_6_) (Kcal/mol)	*E*_cation-π_ (Gr⋅⋅⋅Hydrated M^+^⋅⋅⋅Gr) (Kcal/mol)
K^+^	48 [38]	19.2 [23]	35.4 [22]	−39.6 [43]
Na^+^	54 [38]	28.0 [23]	38.6 [22]	−29.5 [43]
Li^+^	54 [38]	38.3 [23]	47.7 [22]	−25.9 [43]
NH_4_^+^	-	19.3 [23]	-	-

## Data Availability

All the data from this research has been included in this manuscript and Supplementary Materials.

## Supplementary Materials

The following supporting information can be downloaded at: https://www.mdpi.com/article/10.3390/ma16155393/s1, Figure S1: Schematic illustration of the fabrication of a graphene laminar membrane (GLM) by using vapor filtration method with organic support, namely, porous polyethylene terephthalate (PET) membrane; Figure S2: Schematic representation of the electrochemical cells used in this study on the ion transfer across the W/DCE interface supported by different membranes including PET and GLM in the system of W/GLM/DCE system. In cell 1, *x* is the concentration of different chloride salts (R). In cell 2, *y* is the concentration of KNO_3_; Figure S3: The PXRD patterns of (a) PET and (b) the GLM with PET support after immersion inside deionized water for 48 h; Figure S4: *I-V* characteristics of PET obtained in the W/PET/W system respectively containing 0.1 M LiCl, 0.1 M NaCl, 0.1 M KCl, or 0.05 M MgCl_2_ aqueous solution; Figure S5: *I-V* characteristics of GLM in the W/GLM/W system containing 0.1 M KCl or NH_4_Cl aqueous solution; Figure S6: CVs obtained at the PET-supported W/DCE interface by using cell 1 (R is KCl (*x* = 100), NaCl (*x* = 100), LiCl (*x* = 100) or MgCl_2_ (*x* = 50)) under a scan rate (*v*) of 50 mV s^−1^;
