# Supplementary material for: Two-Dimensional Graphene-Based Potassium Channels Built at an Oil/Water Interface
Source: Materials (Basel). 2023 Jul 31;16(15):5393. doi: 10.3390/ma16155393 (PMC10419551; doi:10.3390/ma16155393)
Supplement: Supplementary file 1 [file materials-16-05393-s001.zip › materials-2496754-supplementary.pdf]

materials

MDPI

Supplementary Materials

Two-Dimensional Graphene-Based Potassium Channels Built at an Oil/Water Interface

Xiaoyuan Wang *, Hanhan Yang, Zhenmei Yu, Zengtao Zhang and Yong Chen *

School of Chemical and Environmental Engineering, Shanghai Institute of Technology, Shanghai 201418, China
* Correspondence: wxy18621051556@163.com (X.W.); yongchen@sit.edu.cn (Y.C.);

Contents

1. Preparation and characterization of graphene laminar membrane (GLM) with a PET support..……………………………...…………………….....………..2
2. Ion permeation through GLM in the system of W/GLM/W…..…..……..……. 3
3. Ion transfer across the GLM-supported W/DCE interface in the system of W/GLM/O..………..………………..………..……..………..……...……..…4
4. XRD characterization………..…………………………………….....………..5
5. *I-V* characteristics of PET in the system of W/PET/W……………....…………6
6. *I-V* characteristics of GLM for the permeation of K^+ and NH_4^+…..…………..7
7. CVs obtained at the PET-supported W/DCE interface ………..…….………..8
8. Reference…………………..……………...…………………….....………..9

1. Preparation and Characterization of Graphene Laminar Membrane (GLM) with a PET Support

The detailed setup employed in the fabrication of graphene laminar membrane by the vacuum filtration method is described as Fig. S1 and based on the following steps. Firstly, 2 mL of commercial 2 g/L n-methyl-2-pyrrolidone (NMP) monolayer graphene nanosheets dispersion (flake size ranging from 0.5 to 3.0 µm, Nanjing XFNANO materials Tech Co., Ltd, China) was centrifuged at 5500 rpm with a high-speed centrifuge (TGL-16C, Shanghai Anting Scientific Instrument Factory, Shanghai, China) for 30 minutes. The supernatant in the centrifuge tube was carefully removed and the sediment was re-dispersed in 40 mL isopropanol by sonication for 3 minutes to obtain the graphene monolayered sheet dispersion (~0.1 g/L). Subsequently, as-prepared monolayer graphene nanosheets dispersion (2 mL) was filtered through a porous polyethylene terephthalate (PET) membrane with pore diameter of 0.5 mm, thickness of 5 mm and porosity of ~5 % (Nuclepore track-etch membrane, 47 mm, Nuclepore membrane Company of Haoxia, China) by vacumm filtration pump (HPD-25, Shanghai Mosu Science Equipment Co., Ltd., Shanghai, China) under the pressure of 10000 pa. After that, the PET membrane covered with graphene layers was then dried at 60°C in an oven overnight to obtain a graphene laminar membrane on the PET support, in short GLM. The morphologies and microstructures of GLM were examined by SEM (S-3400N, HITACHI, Japan) and XRD (XRD-6100, SHIMAOZU, Japan).

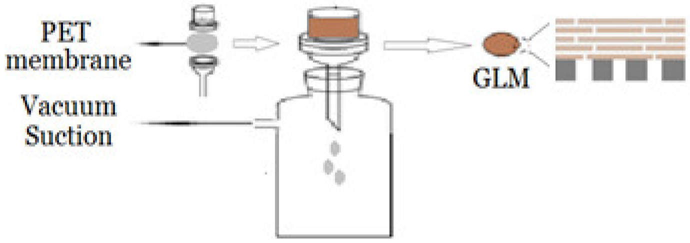

Figure S1. Schematic illustration of the fabrication of a graphene laminar membrane (GLM) by using vapor filtration method with an organic support, namely porous polyethylene terephthalate (PET) membrane.

2. Ion Permeation through GLM in the System of W/GLM/W

Ion permeation measurements were conducted by using a custom-made cell schematically shown in Fig. 1a, where a GLM was placed between two liquid reservoirs to form the W/GLM/W system. The reservoirs were filled with chloride solutions in chosen concentrations, including LiCl, NaCl, KCl, NH_4Cl and $MgCl_2$. (analytical grade, Sinopharm, China) The *I-V* characteristics were measured by using a four-electrode system, namely the opposite electrode (CE) and reference electrode (RE) are platinum wire electrode and Ag/AgCl electrode respectively. CHI660D electrochemical workstation (CHI660D, CHI, USA) and linear sweep voltammetry (LSV) were used for ionic conductance test. The maximum applied voltage was limited to be less than ±0.2 V in order to avoid the possible hysteresis [1]. LSV was set: initial potential: -0.2 V, end potential: 0.2 V, scanning speed: 0.001 V/s, sampling interval: 0.001 V, resting time: 2 s, sensitivity: The 10^{-7}-10^{-5}.

3. Ion Transfer across the GLM-Supported W/DCE Interface in the System of W/GLM/O

The GLM-supported W/DCE interface in the system of W/GLM/O is polarized using four-electrode potentiostats (CHI660D, CHI, USA). The homemade four-electrode electrochemical cell is similar to our previous reports [2-5], where a pair of Ag/AgCl electrodes (one in each phase) work as reference electrodes controlling the potential and a pair of Pt-

wire electrodes (one in each phase) act as counter electrodes measuring the current (Fig. 1b). The working electrode is just the membrane-supported O/W interface, where the ion transfer behaviors are monitored by CV and DPV. All the cell setups (cells 1 and 2) employed for voltammetric ion transfer studies are shown as Fig. S2. In cell 1, the membrane is PET or GLM. In cell 2, the membrane is GLM. The organic phase electrolyte salt was tetrabutylammonium tetraphenylborate (TBATPB) prepared by using tetrabutylammonium chloride (**95%**, TBACl, Aldrich, USA) and sodium tetraphenylborate (**99%,** NaTPB, J&KChemica, China) according to the previous report [6]. **All other chemicals are of analytical grade (Sinopharm, China).** High-resistivity distilled water was used to prepare all aqueous solutions.

Cell 1

Ag	AgCl	1 mM TBACl in 12 mM LiCl	20 mM TBATPB in DCE	*x* mM R	AgCl	Ag

Cell 2

Ag	AgCl	1 mM TBACl in 12 mM LiCl	20 mM TBATPB in DCE	*y* mM KNO_3 in 50 mM $MgCl_2$	AgCl	Ag

Figure S2. Schematic representation of the electrochemical cells used in this study on the ion transfer across the W/DCE interface supported by different membranes including PET and GLM in the system of W/GLM/DCE system. In cell 1, *x* is the concentration of different chloride salts (R). In cell 2, *y* is the concentration of KNO_3.

4. XRD Characterization

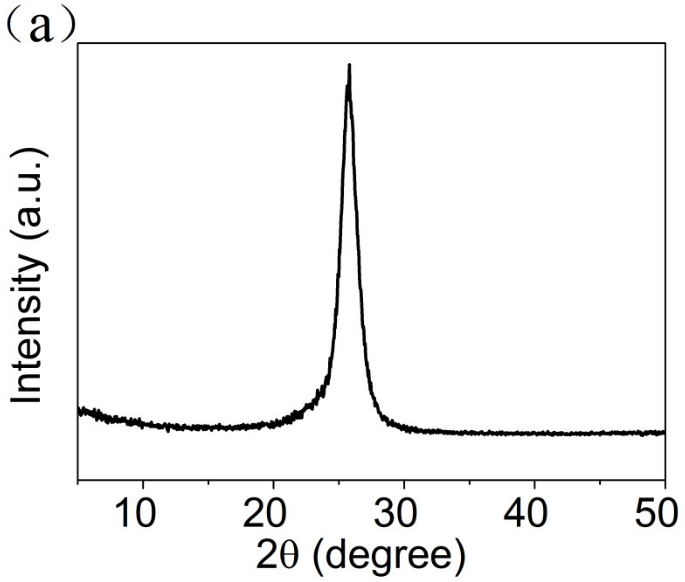

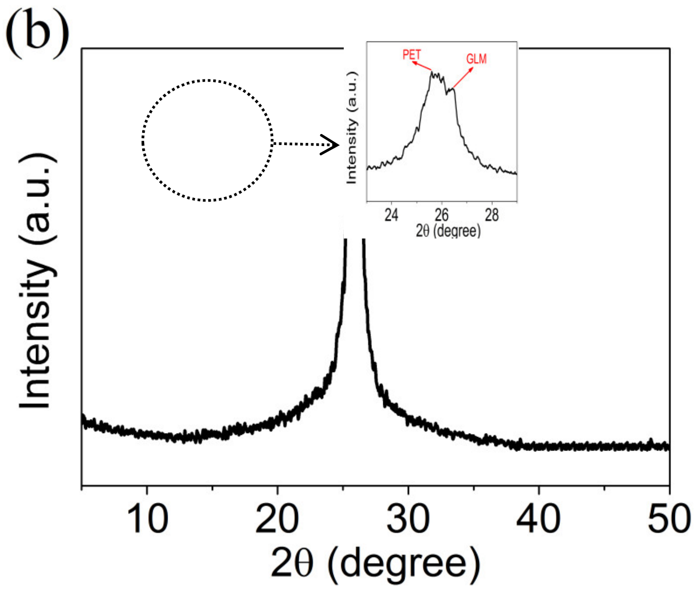

Figure S3. The PXRD patterns of (a) PET and (b) the GLM with PET support after immersion inside deionized water for 48 h.

5. *I-V* Characteristics of PET in the System of W/PET/W

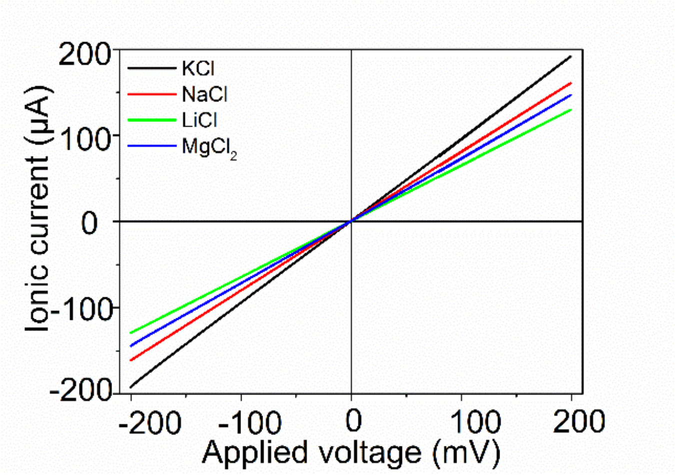

Figure S4. *I-V* characteristics of PET obtained in the W/PET/W system respectively containing 0.1 M LiCl, 0.1 M NaCl, 0.1 M KCl or 0.05 M $MgCl_2$ aqueous solution.

6. *I*-V Characteristics of GLM for the Permeation of K^+ and NH_4^+

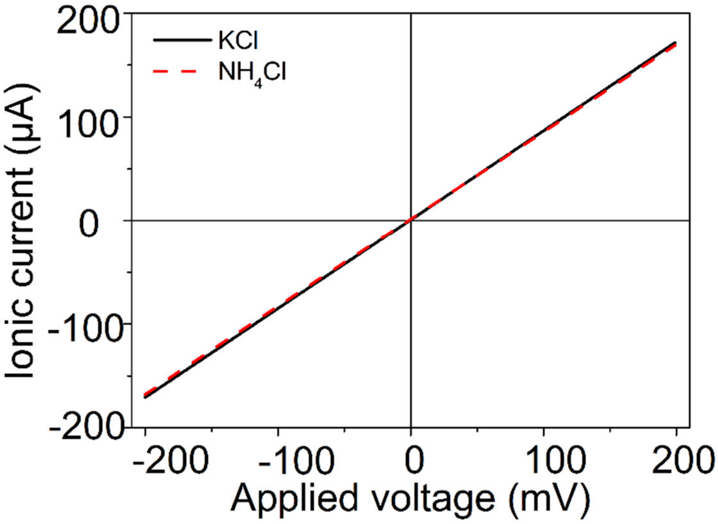

Figure S5. *I-V* characteristics of GLM in the W/GLM/W system containing 0.1 M KCl or NH_4Cl aqueous solution.

7. CVs Obtained at the PET-Supported W/DCE Interface

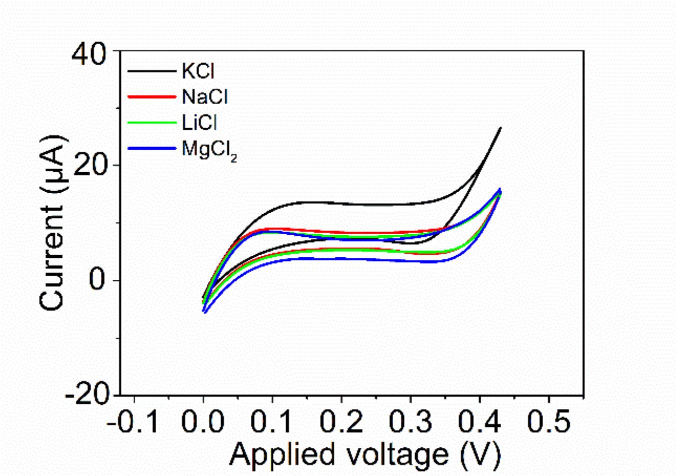

Figure S6. CVs obtained at the PET-supported W/DCE interface by using cell 1 (R is KCl (x=100), NaCl (x=100), LiCl (x=100) or $MgCl_2$ (x=50)) under a scan rate (v) of 50 mV s^{-1}.

8. Reference

[1] Esfandiar, A.; Radha, B.; Wang, F. C.; Yang, Q.; Hu, S.; Garaj, S.; Nair, R. R.; Geim, A. K.; Gopinadhan, K. Size effect in ion transport through angstrom-scale slits. Science. 2017, 358, 511-513. (DOI: https://doi.org/10.1126/science.aan5275)

[2] Chen, Y.; Bian, S.; Gao, K.; Cao, Y.; Wu, H.; Liu, C.; Jiang, X.; Sun, X. Studies on the meso-sized selectivity of a novel organic/inorganic hybrid mesoporous silica membrane. Journal of Membrane Science. 2014, 457, 9-18. (DOI: https://doi.org/10.1016/j.memsci.2014.01.035)

[3] Jiang, X.; Gao, K.; Hu, D.; Wang, H.; Bian, S.; Chen, Y. Ion-transfer voltammetric determination of folic acid at meso-liquid-liquid interface arrays. Analyst. 2015, 140, 2823-2833. (DOI: https://doi.org/10.1039/c4an02011a)

[4] Gao, K.; Jiang, X. H.; Hu, D. P.; Bian, S. J.; Wang, M.; Chen, Y. Impact of an ionic surfactant on the ion transfer behaviors at meso-liquid/liquid interface arrays. Chinese Chemical Letters. 2015, 26, 285-288. (DOI: https://doi.org/10.1016/j.cclet.2014.12.003)

[5] Hu, D.; Wang, H.; Gao, K.; Jiang, X.; Wang, M.; Long, Y.; Chen, Y. Anion transfer across "anion channels" at the liquid/liquid interface modified by anion-exchange membrane. RSC Advances. 2014, 4, 57035-57040. (DOI: https://doi.org/10.1039/C4RA09985K)

[6] Marcus, Y. Thermodynamics of solvation of ions Part 5.-Gibbs free energy of hydration at 298.15 K. Journal of the Chemical Society. Faraday Transactions. 1991, 87, 2995-2999. (DOI: https://doi.org/10.1039/FT9918702995)
